# Increased awareness around an ultra-rare disease can improve diagnosis delays: the French example in primary hyperoxalurias

**DOI:** 10.1186/s13023-026-04463-7

**Published:** 2026-07-04

**Authors:** Lara Cabezas, Aurélie De Mul, Laurence Derain, Romain Pszczolinski, Cindy Castrale, Emmanuel Letavernier, Laurent Mesnard, Dominique Guerrot, Lise Allard, Olivia Boyer, Mélissa Ould Rabah, Isabelle Vrillion, François Nobili, Ariane Zaloszyc, Cécile Acquaviva, Justine Bacchetta, Sandrine Lemoine

**Affiliations:** 1 Service Néphrologie, HTA, dialyse et Explorations fonctionnelles rénales Hopital Edouard Herriot, Hopital Edouard Herriot, Hospices civils de Lyon, 5 place Arsonval, Lyon, 69003 France; 2 Nephrology, Hemodialysis, Apheresis and Kidney Transplantation Department, University Hospital Grenoble, Grenoble, 38000 France; 3 INSERM 1060-CARMEN, université de Lyon, université Lyon 1, Lyon, France; 4 Centre de Référence des Maladies Rénales Rares, Centre de Référence des Maladies Rares du Calcium et du Phosphore, HFME, INSERM 1033 Research Unit, Bron, France; 5 Service de néphrologie-dialyse-transplantation, Hôpitaux universitaires de, Strasbourg, Strasbourg France; 6 Nephrology Department, Saint Martin Hospital, Ramsay Générale de Santé, 18 rue des Roquemonts, Caen, 14000 France; 7 Service des Explorations Fonctionnelles Multidisciplinaires, Hôpital Tenon, Paris, France; 8 Soins Intensifs Néphrologiques et Rein Aigu, hôpital Tenon, Assistance Publique - Hôpitaux de, Paris, Paris, France; 9 Univ Rouen Normandie, INSERM U1096, CHU Rouen, Service de Néphrologie, Rouen, CIC-CRB 1404 France; 10 Pediatric Nephrology Unit, Centre de Référence Maladies Rénales Rares Sorare, Bordeaux University Hospital, Bordeaux, France; 11 Service de Néphrologie Pédiatrique, Centre de Référence des Maladies Rénales Héréditaires de l’Enfant et de l’Adulte (MARHEA), Institut Imagine, Hôpital Necker-Enfants Malades, Université Paris Cité, Paris, France; 12 Explorations Fonctionnelles, Hôpital Universitaire Necker-Enfants Malades, Assistance Publique-Hôpitaux de, Paris (APHP), Paris, France; 13 Service de médecine infantile, Secteur de néphrologie pédiatrique, hôpital d’Enfants de Brabois, CHRU de Nancy, rue du Morvan, Vandœuvre-lès-Nancy, 54500 France; 14 Service de Pédiatrie 2, HôpitalJean Minjoz, CHU de Besançon, Besançon, Jean, Minjoz France; 15 Pédiatrie 1, Hôpital de Hautepierre, CHU de Strasbourg, Université de Strasbourg, 1, avenue Molière, Strasbourg, 67000 France; 16 Service de Biochimie et Biologie Moléculaire, Unité Maladies Héréditaires du Métabolisme, Centre de Biologie et Pathologie Est, Hospices civils de, Lyon, France

**Keywords:** Primary hyperoxaluria, Nephrolithiasis, Nephrocalcinosis, Chronic kidney disease, Genetic kidney disease, Rare diseases

## Abstract

**Background:**

Primary hyperoxalurias (PH) are rare recessive autosomal genetic diseases inducing increased hepatic oxalate production. Apart from genetic background, their prognosis depends mainly on diagnosis delay. We recently reported shorter diagnosis delay in children compared with adults (1.2 (0.1–3.0) *versus* 30 (17–36) years) in a French PH cohort of 52 patients diagnosed between 2015 and 2019. In 2020, the first RNA interfering therapy was approved, leading to increased awareness and communication from reference centres, scientific societies and pharmaceutical companies. Our aim was to evaluate whether diagnosis delay was improved in this global setting.

**Methods:**

We performed a retrospective study including all consecutive patients that received a positive genetic test for PH in our expert genetic laboratory in Lyon between January 2020 and December 2024. We compared outcomes with the historical cohort. Diagnosis delay was defined by the time between the first symptoms and the result of genetic test.

**Results:**

In total, 62 patients (37 children) were included. Forty-two (78%) patients had PH type 1, 5 (8%) had PH2 and 11 (18%) had PH3. The diagnosis delay remained significantly shorter in children as compared to adults (0.75 (1.10) versus 15 (16.21) years, *p* < 0.05), but decreased in both sub-groups when compared to the previous study.

**Conclusions:**

The delay in PH diagnosis in France has been halved over the past five years. This improvement may be associated with recent therapeutic developments and increased awareness of the disease. Earlier diagnosis may enable earlier management, which might ultimately contribute to improved overall prognosis.

## Introduction

Primary hyperoxalurias (PH) are rare autosomal recessive disorders that increase hepatic oxalate production with subsequent oxalate accumulation inducing kidney stones, nephrocalcinosis, chronic kidney disease (CKD), and systemic accumulation (also known as systemic oxalosis) once CKD is present [1, 2]. The prevalence is about 1 to 3 cases per million, but the condition is often underdiagnosed [3]. In 20–50% of cases, severe CKD or recurrence after transplantation occurs before diagnosis [4]. Although there are three types of PH, the most severe form is type 1, with patients being more likely to develop kidney failure (KF) at an earlier age [3]. As a result of progressive and systemic oxalate accumulation, a longer diagnosis delay is well-documented to be associated with progression of CKD to KF [5].

The diagnosis of PH is primarily based on genetic testing following evidence of hyperoxaluria, in the assessment of recurrent, multiple and bilateral lithiasis and/or nephrocalcinosis and/or CKD, especially in young patients from consanguineous families. However, the diagnosis is challenging as nephrologists and urologists must consider measuring urinary oxalate (and plasma oxalate only when glomerular filtration rate, GFR, is below 30 mL/min/1.73 m^2^, because of the risk of false negative when GFR is conserved) [6]. Until recently, there has been a significant lack of awareness regarding this ultra-rare disease. Due to its rarity, hyperoxaluria is not typically considered as a first-line diagnosis, often leading to delays in identifying affected patients. Both the low prevalence of the condition and its non-specific symptoms contribute to this knowledge gap, as many healthcare professionals may not immediately think of it in the context of KF or nephrolithiasis. We previously reported shorter diagnosis delay in children compared with adults (1.2 (0.1–3.0) versus 30 (17–36) years) in a French PH cohort of 52 patients diagnosed between 2015 and 2019 [7].

Moreover, until recently, there were no specific treatments for PH, and management options were limited to supportive measures such as hyperhydration, citrate supplementation, and vitamin B6 [6]. This lack of targeted therapies further compounded the challenges in managing the disease effectively. However, in 2020, the first RNA interfering (RNAi) therapy was approved for PH type 1, namely lumasiran, in Europe and in the USA, for patients of any age and stage of kidney function after the ILLUMINATE industry-sponsored trials [8–10]. A consensus statement from OxalEurope and the European Rare Kidney Disease Reference Network ERKNet was updated in 2023, recommending the use of RNAi therapies for most of PH type 1 patients [6]. This has dramatically modified the management of patients with PH1, and real-life data support its efficacy and safety [11, 12].

As such, this game changer in the management of PH1 led to increased awareness and communication around PH, both from reference centres and scientific societies, but also from pharmaceutical companies. Thus, we hypothesized that diagnosis delay is decreasing in this global setting. Our aim here was to evaluate whether diagnosis delay indeed improved.

## Patients and methods

### Patients

We performed a retrospective study including all patients that received a positive genetic test for PH in our expert genetic laboratory in Lyon between January 2020 and December 2024. Clinical data available at various centers in France were then collected retrospectively including: age, gender, first symptoms and age at onset, age at genetic testing and PH type. The specialty of the practitioner prescribing the genetic test was collected and the type of center where he/she is working (University hospital, other public hospitals, private hospitals … ). Clinical evolution (e.g, kidney transplantation and treatment) was recorded. Patients were categorized as children if they were 18 years or younger at the time of diagnosis.

For genetic testing, PH were diagnosed using massively parallel sequencing (MPS) allowing the simultaneous study of the three genes involved in PH. This method allows the detection of single nucleotide variations located in exons and intron/exon boundaries and the detection of copy number variations.

All procedures were carried out in accordance with the ethical standards, for retrospective studies. The study was conducted according to the guidelines of the Declaration of Helsinki. All individuals gave informed consent before joining the study. This retrospective study was submitted to the local IRB (*Comité d’Ethique des Hospices Civils de Lyon*, under the number 24–5520). The data that support the findings of this study are available from the corresponding author upon reasonable request.

### Endpoints

The primary endpoint was the diagnostic delay, defined as the time between the onset of the first symptoms and the genetic test result. These results were compared with those reported in the previous study by *R. Pszczolinski et al* [7], which used the same definition of diagnostic delay. For patients who received a genetic diagnosis before the onset of symptoms through family screening or prenatal diagnosis, the diagnostic delay was considered to be “0”.

### Statistical analysis

Results are expressed as means with standard deviation and medians with the first and third quartile depending on the distribution. A Wilcoxon test was used for paired comparisons between two groups and Mann-Whitney for unpaired comparisons of two groups. A two-sided *p*-value of <0.05 was considered statistically significant. Statistical analyses were conducted using the R statistical software 4.3.0 [13]. A *p*-value < 0.05 was considered significant for all analyses.

## Results

### Population characteristics

A total of 62 patients were included, 37 (60%) patients were children, 29 (47%) patients were women.

In the adult cohort (Table 1), among the 25 patients, the median age at onset was 22 (15; 38) years. Practitioners requesting the genetic test were mostly nephrologists (*N* = 23, 92%) and were working for the majority in University Hospitals (*N* = 22, 88%). Nineteen (76%) patients had PH type 1, 3 (12%) PH type 2 and 3 (12%) PH type 3. For two patients, the genetic test in Lyon was performed to confirm the diagnosis after another genetic test (exome sequencing). Seventeen (68%) patients had family history of kidney stones, 7 (28%) of whom had a positive genetic test. Six (24%) patients were diagnosed before onset of symptoms, during family screening. Five patients had CKD or KF at the time of diagnosis, fifteen patients had CKD or KF at the time of data collection, and two received kidney transplantation at the time of data collection.

**Table 1:** Adult population characteristics

*N* = 62		
Adult, N(%)		25 (40)
Gender, Male, N(%)		12 (48)
Age at onset, year, Mean (SD)		24.80 (15.64)
Specialist, N(%)	Nephrologist	23 (92)
	Pediatrician	2 (8)
Center type, N(%)	University hospital center	22 (88)
	Clinics and hospital center	2 (8)
	other	1 (4)
Primary hyperoxaluria type, N(%)	1 (*AGXT*)	19 (76)
	2 (*GRHPR*)	3 (12)
	3 (*HOGA1*)	3 (12)
First genetic testing, N(%)	Panel	23 (92)
	Whole Exome Sequencing	1 (4)
	Whole Genome Sequencing	1 (4)
Age at diagnostic, year, Mean (SD)		39.85 (13.37)
Diagnosis delay, year, Mean (SD)		15.05 (16.21)
Family history of kidney stone without genetic testing, N(%)		10 (40)
Family history of kidney stone with genetic testing, N(%)		7 (28)
Family screening, N(%)		6 (24)
Consanguinity, N(%)		6 (24)
First symptom, N(%)	Kidney failure	5 (20)
	Kidney stone	14 (56)
	No symptoms	6 (24)
Symptoms at the time of data collection, N(%)	Kidney stone	17 (68)
	Nephrocalcinosis	2 (8)
	Kidney failure	15 (60)
Age at treatment, year, Mean (SD)		42.29 (13.87)
Treatment, N(%)	Crystallisation inhibitors	19 (76)
	Interferent RNA	12 (48)
	Stiripentol	1 (4)
	B6 vitamin	15 (60)
Kidney transplantation at the time of data collection, N(%)		2 (8)

In the pediatric cohort (Table 2), among the 37 patients, the median age at onset was 0.8 (0.3; 5.3) years. Practitioners requesting the genetic test were mostly pediatricians (*N* = 35, 95%) and were working for the majority in University Hospitals (*N* = 36, 97%). Twenty-seven (73%) patients had PH type 1, 2 (5%) PH type 2 and 8 (22%) PH type 3. For two patients, the genetic test in Lyon was performed to confirm the diagnostic after another genetic test (exome sequencing). Sixteen (43%) patients had family history of kidney stones, 7 (19%) of whom had a positive genetic test. Eight (21.6%) patients were diagnosed before onset of symptoms, during family screening. Eleven patients had CKD or KF at the time of diagnosis, twenty-three patients had CKD or KF at the time of the data collection, and one received kidney transplantation at the time of data collection.

**Table 2:** Children population characteristics

*N* = 62		
Children N(%)		37 (60)
Gender, Male, N(%)		21 (57)
Age at onset, year, Mean (SD)		3.78 (4.83)
Specialist, N(%)	Nephrologist	2 (5)
	Pediatrician	35 (95)
Center type, N(%)	University hospital center	36 (97)
	Clinics and hospital center	1 (3)
	other	0 (0)
Primary hyperoxaluria type, N(%)	1 (*AGXT*)	27 (73)
	2 (*GRHPR*)	2 (5)
	3 (*HOGA1*)	8 (22)
First genetic testing, N(%)	Panel	35 (95)
	Whole Exome Sequencing	1 (3)
	Whole Genome Sequencing	1 (3)
Age at diagnostic, year, Mean (SD)		4.44 (5.04)
Diagnosis delay, year, Mean (SD)		0.75 (1.10)
Family history of kidney stone without genetic testing, N(%)		9 (24)
Family history of kidney stone with genetic testing, N(%)		7 (19)
Family screening, N(%)		8 (22)
Consanguinity, N(%)		7 (19)
First symptom, N(%)	Kidney failure	11 (30)
	Kidney stone	8 (22)
	Nephrocalcinosis	6 (16)
	Hematuria	2 (5)
	Tubular acidosis	1 (3)
	Nycturia	1 (3)
	Mental handicap	1 (3)
	No symptoms	7 (19)
Symptoms at the time of data collection, N(%)	Kidney stone	12 (32)
	Nephrocalcinosis	10 (27)
	Kidney failure	23 (62)
Age at treatment, year, Mean(SD)		4.08 (4.94)
Treatment, N(%)	Crystallisation inhibitors	31 (84)
	Interferent RNA	22 (60)
	Stiripentol	2 (5)
	B6 vitamin	19 (51)
Kidney transplantation at the time of the data collection, N(%)		1 (3)

### Diagnostic delay

The diagnosis delay was still significantly shorter in children as compared to adults (0.25 (0.01; 0.99) versus 8 (1,9; 24,9) years, *p* < 0.05). In the paediatric population, two patients were diagnosed by prenatal testing, one was diagnosed post-mortem (at the age of 9 months old) and eight were diagnosed during a family screening without symptoms.

**Fig. 1:**
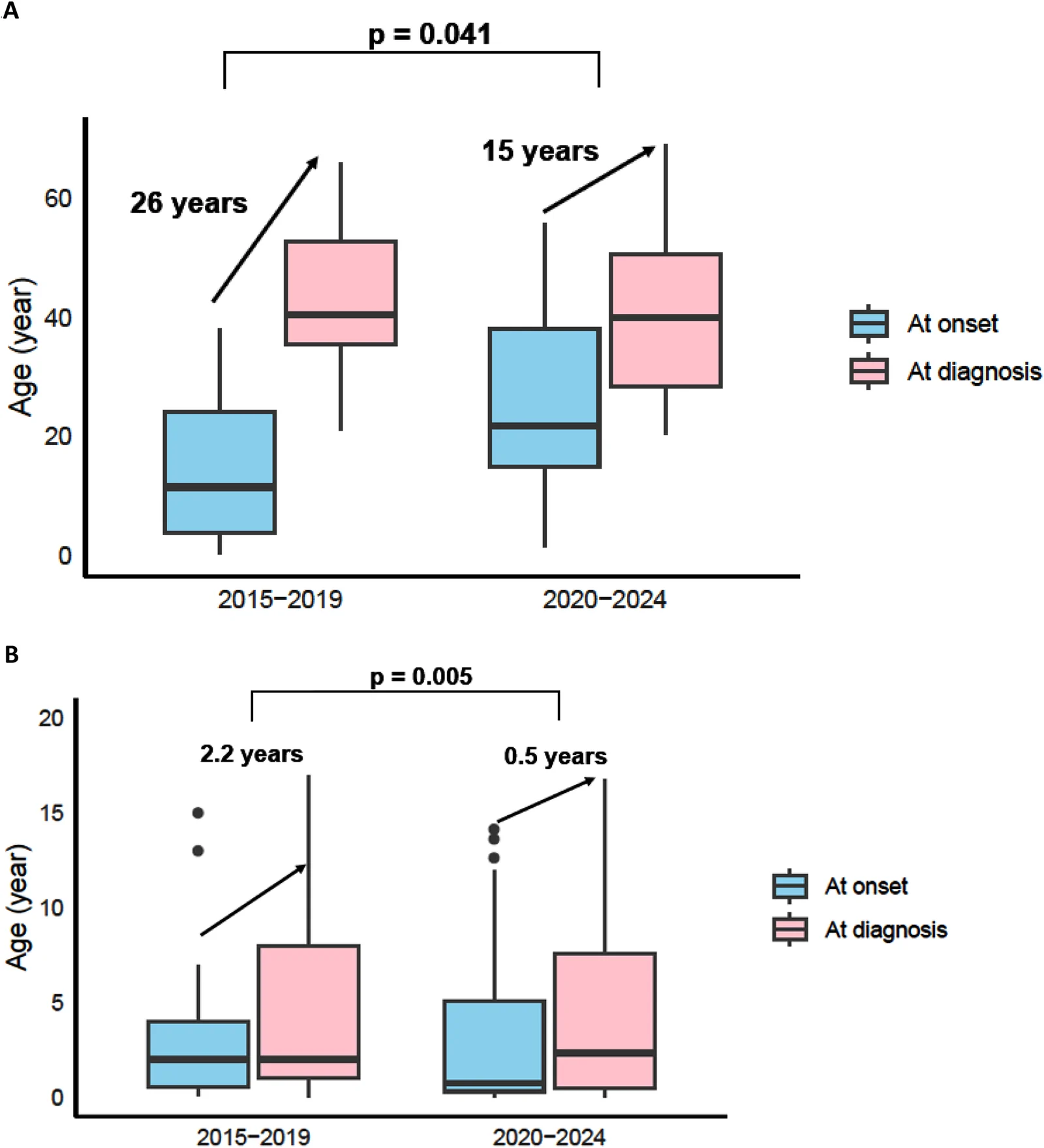
Primary hyperoxaluria diagnosis delay (between age at onset and age at diagnostic) in the cohort of 2015–2019 as compared with our cohort (2020–2024), **A.** In adult, **B.** In children

Comparing diagnosis delay between the current cohort and the 2015–2019 cohort [7], we found that diagnosis delay in adults was significantly lower in the 2020–2024 cohort in adults: 8 (1.9; 24,9) years in the more recent cohort, *versus* 29.8 (16.8; 36.3) years in the 2015–2019 cohort (*p* = 0.041, Fig. 1A). In the paediatric cohort, results were similar, with a significantly lower diagnosis delay in the recent cohort: 0.25 (0.01; 0.99) years *versus* 1.08 (0.19; 2.29) years in the 2015–2019 cohort (*p* = 0.025, Fig. 1B).

## Discussion

When comparing the 2015–2019 and the 2020–2024 cohorts [7], we show a significant improvement in PH diagnosis delay in France since the approval of the first RNAi therapy Lumasiran in 2020 both in children and in adults, that almost halved in the entire cohort.

First, these very encouraging results could be explained by the French organisation and infrastructure around rare diseases, that have been progressively developed and expanded since the first *Plan National Maladies Rares* in 2004, notably with the reference centers for rare diseases and the rare diseases networks: here, the ORKID (ORphan Kidney Disease) network and the Reference Centers for Rare Renal Diseases are very active in the dissemination of information and education on PH. ORKiD is the French national network dedicated to rare kidney diseases, established within the framework of the *Plan National Maladies Rares*. It is organized around national reference centers and regional competence centers that collaborate with research laboratories and patient organizations to improve patient care, research coordination, and knowledge dissemination. While healthcare systems differ between countries, some of these organizational principles—particularly the development of coordinated expert networks and structured referral pathways—may represent transferable approaches to facilitate earlier diagnosis of rare diseases.

Diagnostic pathways for PH often involve multiple specialties—including urology, nephrology, pediatrics, and medical genetics—as well as specialized laboratories. Coordinated care across these specialties may enable earlier referral for genetic testing and contribute to reduced diagnostic delay. This is supported by the SMART study, which demonstrated that a multidisciplinary care model significantly improved the identification of inherited stone diseases, providing a plausible framework for how increased awareness and structured collaboration can translate into earlier genetic diagnosis in real-world practice [14].

Although our findings reflect the organization of the French healthcare system, which benefits from a structured rare disease network and specialized reference centers, diagnostic delay remains a global challenge. A recent nationwide study from Japan on primary hyperoxaluria type 1 reported persistent delays in diagnosis despite increasing awareness of the disease [15]. These findings highlight the importance of coordinated care models, including reference centers and multidisciplinary collaboration, to facilitate patient identification and referral for genetic testing across different healthcare systems.

Guidelines for diagnosis and management have been written in France in 2022 [16], to reinforce the key messages from the recent European guidelines from OxalEurope and ERKNet [6]. In addition to national meetings from the scientific societies (*Société de Néphrologie Pédiatrique*, *Société Française de Néphrologie, Dialyse et Transplantation*) that also communicated around diagnosis and management challenges around PH, the reference centers also participated in dedicated educational webinars, talks and conferences to increase awareness on PH with a focus on nephrologists and paediatricians at the national, European and international levels.

It makes sense that the availability of an effective treatment reduces the feeling of therapeutic helplessness among clinicians upon establishing a diagnosis. Lumasiran (approved in Europe and in the US in 2020) and Nedosiran (approved in the US in 2023) have revolutionized the management of PH1 patients. The European consensus strongly recommends the use of RNAi therapies for most PH1 patients [6]. Recent real-life data on RNAi in routine also support the efficacy and safety of RNAi in PH1 [11, 12]. Novel therapeutic perspectives, considered as real shifts in transplantation strategies, have also been proposed, namely isolated kidney transplantation under RNAi instead of double liver/kidney transplantation [17, 18], that also led to communications in different conferences and subsequently increased global awareness around PH.

Second, even though it will not be possible to quantify the effects of such approaches, this decrease in diagnosis delay could likely be also explained by the information and training provided to healthcare professionals by pharmaceutical companies having developed RNAi therapies. This information can be direct through medical visits from the pharmaceutical companies (including information brochures for physicians and dissemination of guidelines and seminal papers), but also indirect through industry-sponsored webinars and symposiums during local, national, European and international meetings that necessarily increased the global awareness around this ultra-rare disease. Pharmaceutical companies have also developed in France a supportive care program for patients (namely FreeOse) to help patients in their global management, and that may have contributed, however likely to a lesser extent, to improve communication around PH.

However, even though we show a significant decrease in diagnosis delay over time, it still averages 15 years in adults, and we need to collectively continue informing physicians and patients to be able to offer adequate management as early as possible. One future working axis could be to increase awareness around genetic lithiasis (more generally than “only” PH) in urologists, pediatric urologists and general practitioners. Indeed, in case of recurrent and bilateral stones, in a child or in a young adult, especially (but not necessarily) if associated with CKD, nephrocalcinosis, extra-renal symptoms and/or consanguinity, a genetic cause of lithiasis should be ruled out. This is of utmost importance not only for PH but also for other rare diseases that may benefit from targeted therapies, for example cystinuria or lithiasis with increased 1–25 D levels [19–21]. Of note, here, most indications of genetic testing come from university hospitals. Since genetic testing in France is only reimbursed to patients when performed in an hospital setting (but not necessarily a university hospital), it remains debatable whether patients were referred for genetic screening to university hospitals by physicians from private practice or not. In any case, we need to reinforce the links between ambulatory private practice and public hospitals to make earlier diagnoses and to improve the “culture” around rare disease: this is one of the main objectives of the recently launched French *Plan National Maladies Rares* 4 in February 2025. Another point that may facilitate the link between different clinicians and different healthcare structures is the promotion of easy access to molecular diagnostic using “dried blood spot” (DBS).

Moreover, we have diagnosed more patients during the last period, and this is probably due to an easier access to genetic testing combined with increased awareness on the importance of diagnosis early PH1. Of note, in contrast to the 2015–2019 period during which we were sure of the national exhaustivity of genetic testing in Lyon for PH, it was not the case during the 2020–2024 period since more genetic labs have developed “lithiasis panels” on the one hand and because of the development of whole genome analysis with a renal pre-indication validated by the *Plan France Médecine Génomique* on the other hand. The dissemination of genetic testing has also led to an increased number of familial screening in asymptomatic related, from 13% during the previous period to 23% here [7]. We also diagnosed more patients because genetic screening is more largely prescribed, at least in France, in case of “atypical” phenotypes, as reported recently in a premature baby with neonatal tubulopathy and hypomagnesemia who was in fact diagnosed with PH1 by whole genome analysis [22]. Other reports have highlighted cross-linked phenotypes, finding variants in *AGXT* in a patient with a clinical phenotype of Dent syndrome [23].

Doing genetic panels for nephrolithiasis remains important and recommended for young patients, for patients with recurrent disease or bilateral disease who are more likely to have a genetic disease [24]. Indeed, some adult patients from our cohort presented nephrolithiasis since childhood but have not been diagnosed until adulthood. Also, some patients were diagnosed after kidney transplantation for undetermined nephropathy, after a recurrence on the graft that could have been avoided. This supports the concept that genetic testing of PH has a major place in clinical practice in nephrolithiasis but also in undetermined nephropathy in order to prevent recurrence on the graft [24–26]. The CLAFU (*Comité Lithiase de l’Association Française d’Urologie*) recommendations published in 2022 stated that when patients have urinary stone disease, a first line work-up must be systematic as the stone analysis, independently on what stone is found. Second line work-up must be done if there is further disease (further pathology) even after diet modification or if patients have history of recurrent bilateral or multiple stones, nephrocalcinosis, chronic kidney disease, solitary kidney or if patients are young (before 25 years of age) [27]. Genetic tests are included in the second-line work-up and need to be more systematic if there are signs of genetic disease or familial history of kidney stones [27].

Our study presents some limitations. Although the use of a single expert genetic laboratory ensured consistency in diagnostic methods and high diagnostic accuracy, it may have introduced potential selection or detection bias. In addition, the availability and organization of genetic testing have evolved over time, with a progressive decentralization of testing to other laboratories. These changes may have influenced referral patterns and case ascertainment, potentially affecting the observed trends in diagnostic delay. We report on a small cohort due to the rarity of the disease. Biological data were not recorded in our cohort even if biological work-up should be considered in diagnostic process.

## Conclusion

Diagnosis delay in PH in France has decreased over the past five years. This improvement is likely multifactorial, with contributions from increased disease awareness, therapeutic developments, broader availability of genetic testing, and enhanced communication within expert networks, including physicians, scientific societies, national and European reference centers, and pharmaceutical companies. Earlier diagnosis may enable timelier disease management, which could potentially improve overall prognosis in this ultra-rare but severe disease.

## Data Availability

Data and materials are available upon request.

## Abbreviations

PH: Primary Hyperoxaluria
AGXT: Alanine-glyoxylate aminotransferase
GRHPR: Glyoxylate reductase/hydroxypyruvate reductase
HOGA1: 4-hydroxy-2-oxoglutarate aldolase type 1
RNAi: Ribonucleic Acid Interference
ORKID: Orphan Kidney Disease Network
PNDS: Protocole National de Diagnostic et de Soins
